# Technological Advances for Gait and Balance in Normal Pressure Hydrocephalus: A Systematic Review

**DOI:** 10.3390/bioengineering12020135

**Published:** 2025-01-30

**Authors:** Alessandro Zampogna, Martina Patera, Marco Falletti, Giulia Pinola, Francesco Asci, Antonio Suppa

**Affiliations:** 1 Department of Human Neurosciences, Sapienza University of Rome, 00185 Rome, Italy; alessandro.zampogna@uniroma1.it (A.Z.); martina.patera@uniroma1.it (M.P.); marco.falletti@uniroma1.it (M.F.); giulia.pinola@uniroma1.it (G.P.); francesco.asci@uniroma1.it (F.A.); 2 IRCCS Neuromed Institute, 86077 Pozzilli, Italy

**Keywords:** normal pressure hydrocephalus, gait, balance, gait analysis, posturography, wearable sensors

## Abstract

Normal pressure hydrocephalus (NPH) is a recognized cause of reversible cognitive and motor decline, with gait and balance impairments often emerging early. Technologies providing gait and balance measures can aid in early detection, diagnosis, and prognosis of the disease. This systematic review comprehensively discusses previous studies on the instrumental assessment of gait and balance in NPH. A PubMed search following PRISMA guidelines identified studies published between 2000 and 2024 that used laboratory instruments to assess gait and balance in NPH. Studies underwent quality assessment for internal, statistical, and external validity. Methodological details such as motor tasks, instruments, analytical approaches, and main findings were summarized. Overall, this review includes 41 studies on gait and 17 on balance, most of which used observational, cross-sectional designs. These studies employed various tools, such as pressure-sensitive platforms, optoelectronic motion-capture systems, and wearable inertial sensors. Significant differences in kinematic measures of gait and balance have been found in NPH patients compared to healthy controls and individuals with other neurological conditions. Finally, this review explores potential pathophysiological mechanisms underlying the kinematic changes in gait and balance in NPH and emphasizes the absence of longitudinal data, which hinders drawing definitive conclusions for prognostic purposes.

## 1. Introduction

Idiopathic normal pressure hydrocephalus (NPH) is a neurological disorder affecting 3.7% of individuals over 65 and up to 5.9% over 80 [1,2]. It involves ventricular and/or subarachnoid space distension caused by increased cerebrospinal fluid (CSF), despite normal lumbar puncture pressures [3,4,5]. Patients typically present an insidiously progressive gait and balance disorder associated with urinary dysfunction and cognitive decline, often culminating in severe complications and death [5,6]. Temporary symptom relief following a lumbar puncture (tap test) aids diagnosis and predicts response to shunt surgery, which diverts excess CSF to another body site (e.g., peritoneal cavity, heart) [7]. Early treatment can reverse dementia and motor impairments, highlighting the critical need for prompt diagnosis [8].

NPH is underdiagnosed and poorly treated due to several clinical challenges [9]. Its variable presentation can lead to misdiagnosis, as motor signs like bradykinesia and freezing of gait can be mistakenly attributed to parkinsonian syndromes, which require different treatments [10]. Furthermore, while the tap test is commonly used to assess surgical candidates, some patients may benefit from shunt therapy even without obvious clinical motor improvement after acute deliquoration [11,12]. Indeed, although the tap test demonstrates high specificity (75%) and positive predictive value (92%), its low sensitivity (58%) and negative predictive value (37%) limit its accuracy in identifying suitable surgical candidates [13]. Additionally, the absence of reliable predictive measures for long-term surgical outcomes further underscores the need for objective motor assessments to identify NPH objectively and specifically and guide proper treatment decisions early.

Over the last two decades, researchers have increasingly adopted laboratory instruments to objectively assess gait and balance in patients with NPH [12,14,15,16,17,18,19,20,21]. Using different instrumental approaches, such as optoelectronic systems, wearable sensors, and dynamometric platforms and carpets, a large amount of standardized and comparable data have been collected in patients with NPH before and after the tap test and/or shunt surgery. Accordingly, despite not being routinely adopted into daily clinical practice, quantitative measures of gait and balance are already largely available and may help clinicians in the diagnostic and therapeutic management of NPH. Aligned with this observation, a recent meta-analysis examined the responsiveness of gait parameters to CSF drainage in NPH, supporting the use of gait analysis to select patients for shunt surgery [22]. In this systematic review, we have comprehensively summarized previous original studies that adopted laboratory instruments to objectively assess gait and balance in patients with NPH. More in detail, we first report methods and findings of studies assessing gait and balance in NPH objectively through laboratory instruments. Then, we critically examine and discuss the results of our systematic research to provide an up-to-date overview of the knowledge in this field and possible perspectives to improve the clinical management of patients with NPH. Our systematic review expands on previous works by exploring a broad range of analytical and clinical aspects. It analyzes both gait and balance abnormalities in NPH, in comparison not only to healthy controls but also to other neurological conditions. It also provides updated insights into emerging technologies driving advancements in both research and clinical practice. Lastly, it examines the pathophysiological mechanisms underlying gait and balance disorders in NPH.

## 2. Materials and Methods

This systematic review was performed according to the PRISMA statement [23] (Supplementary Materials Table S1).

### 2.1. Search Strategy

Two separate raters (A.Z. and M.P.) independently questioned the PubMed database to find previous original studies adopting laboratory instruments to objectively assess gait and balance in NPH. More in detail, the literature search aimed at the application of different technologies, such as wearable sensors, force platforms and computerized dynamic posturography, to the clinical appraisal of NPH. The queries used for the literature search included the following keywords and their reciprocal combination: (“normal pressure hydrocephalus” or “idiopathic normal pressure hydrocephalus”) and (“gait” or “balance”) and (“sensors” or “accelerometer” or “wearables” or “force platform” or “posturography” or “gait analysis” or “objective assessment”).

### 2.2. Study Selection and Quality Assessment

In this review, we included original studies assessing gait and/or balance in patients with NPH through laboratory instruments. We selected only articles written in English and published from January 2000 to October 2024. The inclusion of studies from 2000 onwards is based on the observation that most research on the topic has been conducted in the past 25 years, likely driven by advancements in technology. We excluded reviews, case reports, and conference proceedings, as well as studies whose full texts are not available or that used non-laboratory instruments, such as stopwatches, activity monitors, and video recordings. First, we examined the titles and abstracts of the articles to exclude duplicate records and off-topic papers. Then, we inspected all the full texts of the identified articles to assess their eligibility based on our inclusion/exclusion criteria. We also checked the reference lists of all the identified articles to collect additional relevant papers possibly missed in the electronic database search.

Following PRISMA guidelines [23], we conducted a quality assessment of the included studies using a 17-item checklist tailored to our topic [24,25,26,27]. The checklist evaluated the internal, statistical, and external validity of the studies (see Supplementary Materials Table S2 for details). The evaluation was independently conducted by two raters (A.Z. and M.P.). Any disagreements were reviewed by two additional raters (M.F. and G.P.) and resolved through collegial discussion to reach a consensus. Each question was rated as “Yes” (2 points), “No” (0 points), or “Not Completely” (1 point), with a maximum score of 34. Studies were categorized as high quality (≥27/34), medium quality (≥20/34), or low quality (<20/34) based on their total score, in accordance with established procedures [27].

## 3. Results

### 3.1. Search Results and Study Selection

**Figure 1:**
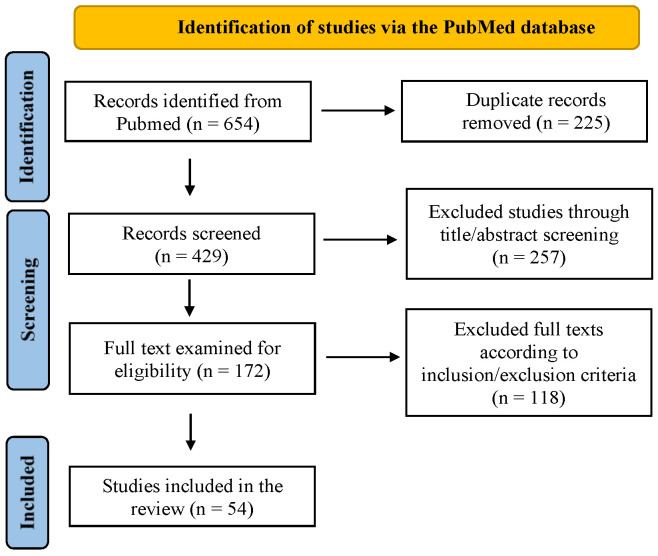
Flow diagram for the selection of studies.

Our query retrieved 654 potentially eligible articles for the systematic review. After removing 225 duplicates, we screened titles and abstracts, excluding 257 articles (e.g., off-topic articles, pre-2000 studies, conference proceedings, and non-laboratory studies). Following a full-text review based on the inclusion/exclusion criteria, we excluded 118 articles. Manual reference searches identified two relevant studies. This process resulted in the inclusion of 54 articles: 41 on gait assessment and 17 on balance in NPH, with 4 addressing both. Figure 1 presents a flow diagram summarizing the search and screening strategy.

### 3.2. Gait in NPH

Table 1 summarizes the main methods and findings of the 41 selected articles focused on the objective assessment of gait through laboratory instruments.

#### 3.2.1. Methodological Approach

The size and sample population characteristics were variable across the 41 included studies in the objective evaluation of gait in NPH. Twenty-seven studies enrolled a limited number of patients with NPH with a size range of 4 to 42 patients [12,15,18,19,28,29,31,33,35,36,37,38,40,42,44,45,46,48,49,50,51,52,55,56,57,58,61], whereas only 14 studies included a large number of patients with a size range of 50 to 97 patients [14,16,17,30,34,39,41,43,47,53,54,59,60]. The enrolled patients with NPH overall showed a mean age of 75.4 ± 2.23 years. Twenty-three studies included a control group of age-matched healthy subjects (HS) [12,14,18,19,28,34,35,36,38,40,42,44,45,47,49,50,53,55,56,57,58,59,61]. Also, some authors (i.e., 6 studies) compared patients with NPH and those suffering from other neurological disorders, such as Parkinson’s disease (PD) [18,29,38,56,57], progressive supranuclear paralysis (PSP) [29,35], dementia, and other conditions responsible for hydrocephalus-like changes in gait [29,32,38].

Most studies (i.e., 31) assessed gait objectively before and after acute and/or chronic deliquoration [12,14,15,16,17,18,19,28,29,30,31,32,33,34,36,37,38,40,41,42,44,45,47,48,49,51,52,53,54,58,60]. More in detail, 26 studies used the tap test or transitory CSF drainage to assess the effects of acute deliquoration on gait in NPH [12,14,15,16,17,18,19,28,29,30,31,32,34,36,37,38,40,41,42,45,47,48,51,52,54,60]. Among these, seven studies also assessed gait changes due to chronic deliquoration in patients with NPH that underwent shunt surgery [12,15,19,37,38,40,41,48,49,51,52,53,58]. Only a few authors investigated the isolated effects of shunt surgery [33,44,49,53,58]. Lastly, 10 studies examined baseline gait parameters without considering the effects of deliquoration [35,39,43]. Among studies that used the tap test or transitory CSF drainage, most (i.e., 22 out of 26) specified that acute deliquoration involved the removal of at least 30 mL CSF (CSF range: 30–50 mL) [12,14,15,16,18,28,29,30,31,32,34,36,37,38,40,41,42,45,47,51,52,60], whereas four studies did not clarify this point [17,19,48,54].

When considering the effects of the tap test or transitory CSF drainage, some authors analyzed gait a few hours after the acute deliquoration (i.e., 1 to 8 h) [14,15,31,34,37,48,51,54], whereas others adopted a prolonged time for the re-assessment (i.e., 24 to 96 h) [12,16,17,18,19,28,29,30,31,32,36,38,40,41,42,45,47,48,52,54,60]. Similarly, patients with NPH who underwent shunt therapy were assessed 1 week to 6 months after surgery [15,19,37,40,41,48,51,52,58]. Only two articles did not specify the timing for clinical re-assessment after shunt surgery [49,53].

Concerning the motor tasks adopted to assess gait in NPH, most studies used free-speed locomotion along paths with different lengths (i.e., 3 to 18 m), including the timed up-and-go (TUG) test [14,16,18,19,28,29,30,32,33,34,35,36,37,38,39,40,41,42,44,45,46,47,48,49,50,51,52,53,54,55,56,57,58,59,60,61]. Two articles collected data from 72 h of home-monitoring of patients and compared them with an experimental evaluation [50,58]. A few authors also assessed the effect of external cues [18] and cognitive functions (i.e., dual task) on gait [29,31,32,35,53,59,61]. Lastly, three studies analyzed possible changes in gait parameters with different walking velocities (i.e., preferred and maximal velocity) [31,59,61].

Concerning the objective evaluation of gait in NPH, we identified three main instrumental settings: i. pressure-sensitive systems [12,14,15,29,31,33,35,36,37,41,42,48,53,54,59,60], ii. wearable inertial sensors [16,19,30,34,38,39,40,44,45,47,48,49,50,51,52,57,58,61], and iii. optoelectronic systems [17,18,28,29,32,33,43,44,46,55,56]. Only a few authors adopted additional devices, such as a knee goniometer, optical infrared sensors, or punched-out peaks under shoe soles, imprinting footmarks on a draft paper, in combination with a pressure-sensitive or optoelectronic system [14,18,28,49]. Wearable inertial sensors included one to three tri-axial accelerometers and smartphones equipped with inertial sensing systems, positioned mostly on the waist, abdomen, and lower limbs [16,19,30,34,38,39,40,44,45,49,50,51,52,57,58,61], and rarely on the head and upper limbs [45,50,58,61]. Lastly, one author applied machine learning algorithms to inertial gait data, using a data-driven approach to classify pathological strides [50].

Key outcome measures of the selected studies included TUG duration [36,40,46,52,57]; gait cycle phases [12,14,15,16,18,28,29,34,37,42,43,48,50,51,52,53,55,57,58,59,60]; spatiotemporal kinematic parameters such as cadence, velocity, stride/step length/time/width/height, and gait variability indices [12,14,15,16,17,18,19,28,29,30,31,32,33,34,35,37,39,41,42,43,44,45,46,48,49,50,51,52,53,55,57,58,59,60]; plantar pressure variability[48]; body segment fluctuation [56]; and joint angles/ROM [14,15,18,28,33,37,42,47,50,51,52,57,58,60,61].

#### 3.2.2. Main Findings

Compared to HS, patients with NPH had longer TUG test times, mainly due to an extended gait cycle, with notable prolongation of the double-limb support phase and shortened swing and single-limb support phases [12,14,18,28,34,38,40,42,50,53,57,59]. They also exhibited reduced cadence, velocity, stride length, and step length/width/height compared to HS [14,18,19,28,34,35,42,44,50,53,55,57,58,59]. The stride length/time coefficient of variation (CV) was increased [18,28,35,42,53,59], while the step time/width CV was reduced [18,19,35]. Additionally, patients had higher plantar pressure variability [48], altered heel-height variability [49], and significant fluctuations in all body segments [56] compared to HS. Regarding joint angles, increased toe-out angle and reduced range of motion in limb joints were observed [14,18,28,42,58,61]. Finally, gait parameters correlated with frontal cognitive functions [42,51], clinical scores [52,59], and radiological changes (e.g., temporal horn value, Evans index) [31,33,54].

Compared to patients with parkinsonian syndromes, those with NPH exhibited lower gait velocity, stride length, step height, gait variability, and joint range of motion, but higher step width and body fluctuation indexes than patients with PD or PSP [18,35,56,57]. Additionally, NPH patients experienced less gait impairment during dual-task conditions compared to those with PSP [35]. By contrast, Allali and colleagues found no significant differences in baseline spatiotemporal gait parameters between NPH patients and those with other conditions mimicking NPH, such as vascular, frontotemporal, and alcoholic dementia, under either single- or dual-task conditions [29].

Concerning the effects of acute and chronic deliquoration, most gait parameters, including the duration of the TUG test, gait cycle phases, and kinematic spatiotemporal parameters, improved after the tap test and/or shunt surgery in NPH [12,14,15,16,17,18,19,28,29,30,31,32,33,34,36,37,38,41,42,44,45,49,51,52,52,57,58,60]. The improvement in gait kinematics was higher in patients with NPH presenting a frontal-like gait than those with a parkinsonian-like gait [17]. The extent of parameter response to deliquoration was significantly correlated with the patients' Evans index (i.e., a radiological marker of ventricular volumes) [31,33]. A few authors reported a greater improvement in specific gait parameters, such as gait velocity, stance phase duration, stride length, and base width, following shunt surgery compared to the tap test [12,37,51]. Changes in the double-limb support phase duration, gait velocity, stride length, and cadence after acute deliquoration significantly correlated with the response to the shunt surgery [12,31,51]. Among these parameters, gait velocity and stride length were proposed as the most sensitive response marker to acute deliquoration in NPH [28,58]. Changes in step width were the most discriminative parameter among patients with NPH and those with mimicking conditions [29]. However, one study found that baseline spatiotemporal gait parameters could not differentiate between NPH patients who improved after acute deliquoration and those who did not [30]. Based on kinematic measures, some authors calculated specific indices and thresholds to differentiate NPH gait from that of HS. In particular, fluctuations in trunk tri-axial accelerations, reduced stride length and gait velocity, and an increase in the double support phase and stride length CV were significantly associated with the NPH pathological gait [47,53]. Some authors also applied machine learning algorithms for the automatic classification of stride profiles in patients with NPH [50].

**Figure 2:**
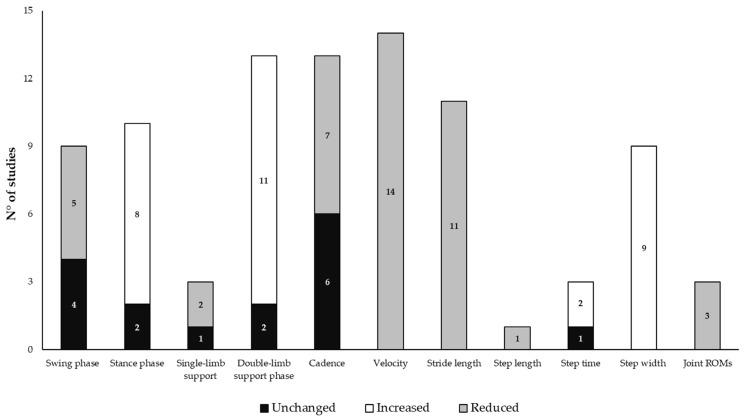
Consistency of studies on the main findings of gait changes when comparing patients with normal pressure hydrocephalus and healthy subjects. Note: measures varied across studies; the main methods and findings of the 17 selected articles focused on the objective assessment of balance through laboratory instruments are summarized.

Regarding the time of assessment after the tap test, a few authors showed that the main changes in spatiotemporal gait parameters in NPH occurred after 24–72 h after acute deliquoration, thus possibly demonstrating the existence of some false-negative results within the first 24 h [16,31,41,51]. Table 2 summarizes changes in the main kinematic measures of gait in patients with NPH before and after acute and/or chronic deliquoration, while Figure 2 displays the consistency of studies on the main findings when comparing patients to HS.

### 3.3. Balance in NPH

Table 3 summarizes the main methods and findings of the 17 selected articles focused on the objective assessment of balance through laboratory instruments. 

#### 3.3.1. Methodological Approach

Most of the studies concerning balance involved a limited number of NPH patients. In detail, 16 studies involved small cohorts ranging from 9 to 40 subjects, whereas only one study presented a larger cohort with a sample of 56 NPH patients [71]. The enrolled patients’ mean age was 72.2 ± 5.3 years.

Twelve studies included a control group of age-matched HS [20,21,55,57,61,62,63,64,65,66,68,69,70,72]. In contrast, six articles compared NPH patients to those affected by other neurological disorders, such as PD [57,72], ventriculomegaly [68], brain atrophy [62,64,65], and subcortical arteriosclerotic encephalopathy [20].

The effects of CSF drainage on balance performance were assessed in nine articles. More in detail, three studies reassessed the patients after isolated acute deliquoration [67,70,71], seven after shunt surgery [15,20,62,65,66,68,69], and two after both procedures [15,68]. Lastly, seven articles assessed baseline changes of balance in NPH patients without investigating the effects of CSF drainage [21,55,57,61,63,64,72].

Among the evaluated effects of acute deliquoration, postural abilities were examined less than 24 h [15,68,71] or 72 h [69] after the tap test procedure. Studies evaluating the effects of chronic deliquoration reassessed patients 1 week [65,69] or 3 months [15,20,66,68] after the shunt surgery. Only a few papers did not report any information about the time of assessment after acute or chronic deliquoration [62,67].

Concerning the motor tasks adopted to assess balance, postural abilities were mostly assessed during the upright stance or, alternatively, during a 5–10 m walking test [55,68] or the pull test [70]. Several authors explored the impact of sensory stimuli, such as vision, by having participants close their eyes [20,61,62,63,64,65,66,67,68], and proprioception, by asking patients to walk on foam surfaces [68]. A few others evaluated voluntary multidirectional leaning tasks [69,72].

Regarding the experimental setup, force platforms were the most adopted instruments to assess balance [20,21,62,63,64,65,66,67,69,72]. Alternative solutions included inertial sensors [15,57,68,70] or optoelectronic systems [55]. The main outcome measures described the main changes in center of pressure (COP) and center of mass (COM), including anteroposterior (AP) and mediolateral (ML) displacements [20,21,57,61,63,65,68]; mean COM/COP displacements, such as the locus length or the sway radius [61,62,64,65,69,72]; the sway area [15,20,57,61,62,65,69,72]; AP and ML sway velocities [20,21,55,57,63,68,71]; mean sway velocity [57,70,71]; and the stability area [21,69,71,72]. Other parameters reported were AP and ML maximum leaning distances during voluntary multidirectional leaning [69,72], AP and ML distances between COM and the base of support (COM-BOS distance) [55], and reaction times and COM acceleration during the pull test [70].

#### 3.3.2. Main Findings

When examining baseline changes in balance, patients with NPH showed increased AP and ML sway [20,21,57,61,63], sway area [20,57,62,64,65,69,72], sway radius [62,64,65], and locus length [69,72] during quiet standing, as well as reduced AP and ML leaning distances during multidirectional leaning tests compared to HS [69,72]. Moreover, patients with NPH were characterized by higher sway velocities [20,21,57,63,68], lower COP stability areas [21,69,72], and worse balance performances under different sensory conditions (i.e., sensory organization test scores) than HS [66,67].

Concerning the comparison with other pathological conditions, balance assessment disclosed significant differences between patients with NPH and those affected by subcortical arteriosclerotic encephalopathy and brain atrophy. More in detail, compared to subcortical arteriosclerotic encephalopathy, patients with NPH showed higher AP displacement and lower AP/ML ratio and AP sway velocity when standing with feet together and eyes closed [20]. Also, NPH patients showed higher sway area/radius than patients with brain atrophy [64,65] and lower sway velocity during gait than patients with ventriculomegaly [68]. Compared to PD, NPH patients presented increased sway area [57,72], root mean square sway, and ML displacement [57]; reduced stability area; and increased locus length per unit area [72].

Regarding the effects of acute and chronic deliquoration, most studies consistently reported a significant improvement in balance parameters in patients with NPH. When considering acute deliquoration, Abram et al. reported increased composite sensory organization test scores (i.e., 2,4,5), associated with improved somatosensory and visual performances [67]. Among many parameters, authors reported improvement in the AP sway angle [68] and sway velocities [68,71], and a reduction in the base of support [71] during quiet standing, plus reduced AP/ML sway velocities during gait [68]. Similarly, patients with NPH who had undergone shunt surgery showed reduced sway area [20,62,65,68,69], sway radius [62,65], COP length trajectory [65], AP displacement [20], and increased stability area and leaning distances in the post-surgical period [69]. Conversely, only a minority of authors did not report any significant changes in postural abilities in patients with NPH after acute or chronic deliquoration [15,21,66].

Finally, several authors investigated the impact of visual deprivation on balance performance and obtained controversial results. In fact, in different studies, NPH patients showed both worse [20,61,63,64,66] and better postural performance [62,65,68] after eye closure compared to HS. The impact of eye closure after acute and chronic deliquoration was equally controversial: some authors reported improvement in postural response with eyes closed after CSF removal [66,67], while others described significant worsening [62,65,68].

**Figure 3:**
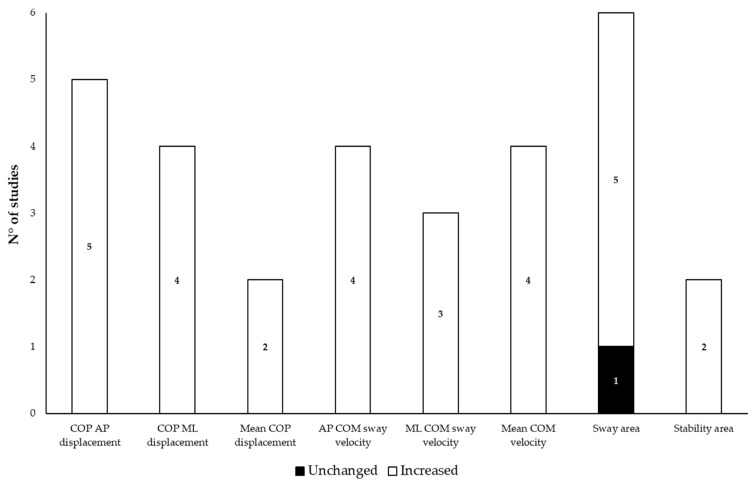
Consistency of studies on the main findings of balance changes when comparing patients with normal pressure hydrocephalus and healthy subjects. Note: measures varied across studies; therefore, the displayed counts differ from the total studies.

Table 4 summarizes the main kinematic measures of balance in patients with NPH before and after acute and/or chronic deliquoration, while Figure 3 displays the consistency of studies on the main balance findings when comparing patients to HS.

### 3.4. Quality Assessment

Among the studies focused on gait evaluation, 17 (41%) were categorized as high quality, 23 (56%) as medium quality, and 1 (3%) as low quality. In comparison, studies assessing balance showed 8 articles (47%) classified as high quality and 9 (53%) as medium quality, with no studies falling into the low-quality category. Detailed results of the quality assessment for each study included in this review are provided in Supplementary Materials Table S2.

## 4. Discussion

Following PRISMA guidelines, this systematic review provides a concise overview of the current research on instrumental gait and balance assessments in NPH. We have demonstrated that a specific pattern of kinematic alterations affecting gait and balance can be instrumentally identified in patients with NPH compared to HS or other neurological conditions considered in the differential diagnosis. The adopted methodological transparency facilitates the replication of the review and enables readers to critically evaluate the strengths and limitations of instrumental approaches in this field. Additionally, a comprehensive quality assessment of the included studies minimizes potential biases and overinterpretation, offering a clear perspective on how existing findings might inform the clinical management of NPH.

### 4.1. Gait and Balance in NPH: Instrumental Assessment

While significant progress has been made in instrumentally characterizing gait and balance in NPH, substantial methodological heterogeneity is evident in the literature. When considering adopted technologies, different authors used several tools, including pressure-sensitive platforms, optoelectronic motion-capture systems, and wearable inertial sensors, each with specific strengths and limitations [73]. Platforms provide precise force and pressure data during both static and dynamic motor tasks but are limited to laboratory settings and can be relatively costly [74]. Similarly, optoelectronic systems capture detailed spatial–temporal parameters and joint kinematics with high accuracy but require complex setups [75]. Though less precise, wearable sensors measure spatial–temporal parameters of balance and gait and are highly portable, thus potentially enabling long-term and real-world monitoring of movement [74]. Given abnormal intracranial pressure fluctuations in NPH, as shown through intraventricular devices [76], wearable sensors could be a valuable research tool for long-term movement monitoring in real-life conditions, benefitting from their ease of use and affordability [77]. In addition to the instrumental heterogeneity, there is variability in the motor tasks, including pathways of different lengths to assess gait at varying speeds and postural exercises under different visual conditions to evaluate balance. Moreover, an additional methodological source of variability is the timing of gait and balance assessments following CSF drainage. Only a minority of authors have adhered to current recommendations, which advocate for multiple evaluations within the first week after acute deliquoration, including assessments within 24 h (i.e., 2–4 h), the following day, and up to 7 days after [5,78,79]. Lastly, data analysis approaches have also varied. While some studies have performed classic statistical comparisons between groups, other authors have applied advanced machine learning methods, suggesting potential applications [50,80]. Machine learning techniques offer promising opportunities for improving data analysis in NPH by analyzing and merging extensive datasets from clinical, imaging, and sensor measurements, potentially helping to identify patterns and predict outcomes with greater accuracy [81,82]. Overall, the high variability in assessment tools and experimental protocols affects the comparability and reproducibility across investigations. This underscores the need for standardized approaches, as also emphasized by a recent meta-analysis addressing the topic of gait analysis in NPH [22].

When specifically focusing on gait, the reviewed studies identified several abnormalities in spatiotemporal and kinematic parameters in patients with NPH, such as prolonged double-limb support phase and increased step width, associated with reduced gait velocity and stride length [22]. Some discrepancies exist among studies when examining cadence [18,42,57], although a recent meta-analysis provided statistical evidence of a significant reduction in this feature in patients with NPH compared to HS [22]. Overall, most studies identify a set of kinematic gait alterations that differentiate patients with NPH from HS and individuals with other neurological conditions associated with hypokinetic gait, such as PD and atypical parkinsonism [14,18,19,28,34,35,42,45,50,53,55,57,58,59]. While these kinematic alterations alone are not specific to a distinct disease, their combination may suggest a possible movement profile characteristic of NPH. This composite gait pattern could aid in the disease assessment and management of patients, providing objective measures of movement impairment that may help guide treatment decisions and monitor disease progression over time [61]. Most kinematic parameters partially improved following acute and chronic CSF drainage, supporting their potential utility also for treatment outcome monitoring. In particular, changes in gait velocity appear to be the most consistently reported and sensitive measure in NPH patients who respond positively to CSF drainage [22]. Gait velocity is a widely recognized measure of motor performance and functional mobility, which is particularly valuable in the assessment of neurological disorders and ageing-related conditions [83]. Generally, in neurological diseases, reduced gait velocity correlates with disease severity, fall risk, and decreased quality of life [84,85]. Similarly, when examining balance, increased anteroposterior and mediolateral sway, larger sway areas, and reduced leaning distances partially improved after acute and chronic deliquoration. Partial improvement in balance after shunting may reduce fall risk in NPH patients [39]. However, balance relies on complex brain networks, and the suboptimal response to interventions is due to several factors [74]. Cognitive impairments, common in these patients, often lead to poor responses to CSF drainage [86]. Additionally, peripheral and/or central vestibular dysfunctions contribute to postural instability and reduce the effectiveness of therapeutic interventions [39]. Overall, the incomplete recovery of gait and balance abnormalities suggests that other factors beyond intracranial pressure, possibly irreversible, may contribute to motor dysfunction in NPH [87].

### 4.2. Pathophysiological Insights from Gait and Balance Kinematics in NPH

Despite heterogeneous methodological approaches, studies highlighted a distinctive pattern of walking and balance impairments in patients with NPH. Specifically, gait in NPH is characterized by small, “magnetic” steps (i.e., shuffling gait) with a broad base of support, reduced walking speed, and increased stride length variability. Both static and dynamic postural control are compromised, as evidenced by heightened multidirectional oscillations during upright stance and diminished functional stability during body excursions. Notably, patients with NPH often exhibit extra-rotated feet during gait, resembling a “duck-footed” walking pattern. This adaptation likely serves as a compensatory mechanism to enhance stability by widening the base of support and prolonging the double-limb support phase. Pathophysiologically, several factors, including hypoperfusion, glymphatic impairment, disturbance of metabolism, astrogliosis, neuroinflammation, and blood–brain barrier disruption, jointly participate in NPH clinical development and kinematic abnormalities [88]. The mechanical stretching and compression of neural fibers adjacent to the lateral ventricles, potentially extending to brainstem structures such as the pedunculopontine nucleus, has been suggested as a possible trigger factor [89]. Previous studies have demonstrated reduced cerebral perfusion in different key regions involved in gait and balance, including the basal ganglia, frontal lobes, and temporal lobes [78,90,91,92,93], which is possibly linked to the mechanical stress on the parenchyma and blood vessels induced by ventricular enlargement. In line with this hypothesis, deliquoration could restore cerebral perfusion in these areas, leading to clinical improvement in patients with NPH [93]. Most studies reviewed herein have demonstrated significant changes in various spatiotemporal gait parameters following both acute and chronic deliquoration in NPH. Notably, some of these changes correlated with ventricular volumes and the clinical phenotype of gait impairment, with frontal-like dysfunction showing greater responsiveness to deliquoration [17,33,53]. Consequently, quantitative gait parameter changes after acute deliquoration may serve as a sensitive tool for monitoring therapeutic outcomes, complementing their utility in the diagnostic process by reflecting underlying brain abnormalities and differing therapeutic responses. Indeed, identifying the neurophysiological changes and brain regions associated with abnormal gait and balance kinematics could help clinicians better interpret instrumental findings, supporting more accurate differential diagnoses, therapeutic interventions, and tailored rehabilitation strategies.

A noteworthy clinical challenge lies in the differential diagnosis of NPH and neurodegenerative disorders that present with overlapping symptoms, such as parkinsonian syndromes and other neurodegenerative conditions. Patients with NPH often exhibit parkinsonian signs, including bradykinesia, rigidity, and a small-step gait [89]. The differential diagnosis between NPH and other parkinsonian disorders is challenging, due to the similar clinical presentation and the transient gait improvement observed in some cases of vascular parkinsonism after acute deliquoration [79]. Parkinsonian features in NPH likely stem from hypoperfusion of the basal ganglia rather than classical dopaminergic cell loss [92], as suggested by abnormalities in nigrostriatal dopaminergic imaging (e.g., DAT scans) and the lack of response to levodopa treatment [94,95,96]. Other pathological conditions presenting radiological or clinical similarities to NPH are vascular or neurodegenerative dementias, such as subcortical arteriosclerotic encephalopathy, brain atrophy, or Alzheimer’s disease. Concerning the latter, NPH can sometimes coexist with Alzheimer’s disease, likely due to the similar age of onset and potential shared pathophysiological mechanisms. It has been hypothesized that abnormal CSF production and turnover in NPH may impair the clearance of amyloid–beta peptides and tau protein from the brain interstitial fluid space [97], a process that is further exacerbated by reduced cerebral venous compliance [98]. Together, these factors could contribute to neuronal dysfunction and reduced survival, potentially initiating a neurodegenerative cascade [97]. However, these remain hypotheses that are not yet supported by sufficient data, and the studies included in this review do not provide comparisons between NPH and Alzheimer’s disease patients.

As a final observation, this review confirms controversies about visual deprivation on balance in NPH. Indeed, some studies have reported that postural performance in NPH paradoxically does not deteriorate with eye closure [99]. The neural control of postural stability depends on the integration of somatosensory, vestibular, and visual inputs, and maintaining equilibrium requires coordinating these systems to produce motor strategies that maintain the COM in a stable range to prevent falls [70]. It has been suggested that balance in patients with NPH does not worsen following eye closure owing to possible hyper-compensation by specific neural networks in response to the baseline impairment of postural control. In line with this hypothesis, increased bilateral activation of the supplementary motor area has been observed in patients who do not exhibit worsened performance following visual deprivation [99]. A similar hypothesis has been suggested to explain the observation that patients with NPH demonstrate improved gait velocity during dual motor tasks compared to PSP patients, likely due to compensatory hyperactivity of the prefrontal cortex [35].

Although conducted according to rigorous methodological standards, this systematic review has some limitations that must be acknowledged. As previously highlighted, the primary challenge in reaching clear conclusions stems from the significant heterogeneity in study designs, methodologies, and patient populations, which complicates the comparability and generalizability of the findings. Variations in how gait and balance are assessed, differences in patient demographics, and diverse treatment protocols make it difficult to draw consistent conclusions across studies. Moreover, many studies rely on cross-sectional or retrospective designs, which limit the ability to establish causality or assess long-term outcomes effectively. Finally, there is a lack of high-quality, prospective studies that explore the long-term prognostic value of instrumental measures of gait and balance abnormalities in NPH patients.

## 5. Conclusions

This systematic review analyses the literature on instrumental gait and balance assessment in NPH, focusing on studies using laboratory equipment and wearable technologies. Numerous studies have identified quantitative measures of gait and balance disorders that could aid in early detection and differential diagnosis of NPH. These studies also highlight how specific kinematic measures change in response to common CSF drainage therapies. Although no single kinematic measure is uniquely associated with NPH, a complex pattern of gait and balance abnormalities appears to be more distinctive of the condition, offering potential value in clinical management. However, evidence remains limited regarding reliable predictive markers for shunt surgery outcomes. Most studies have short-term follow-ups, hindering a full understanding of how gait and balance improvements correlate with long-term recovery or quality of life. Further key limitations in available studies include uneven group sizes, inconsistencies in study design and preparation, and challenges in selecting homogeneous groups of patients. Also, in terms of analytical approaches, reliance on traditional statistical methods, the requirement for balanced data sets, and the underutilization of AI for advanced classification and predictive analysis remain notable constraints. More research is therefore needed to establish how kinematic measures of balance and gait can predict surgical success in patients with NPH. Future studies should adopt standardized protocols and include larger, more diverse cohorts to improve the reliability and clinical relevance of the findings. Moreover, efforts should aim to bridge the gap between traditional methods and modern approaches, such as machine learning, to enable meaningful comparisons across periods and populations. Developing novel computational parameters, like fractal dimensions reflecting gait uniformity, could also offer significant potential for deeper insights and improved population monitoring.

## Figures and Tables

**Table 1:** Instrumental evaluation of gait in normal pressure hydrocephalus.

Study	NoS	CSF Drainage	Assessment Time After Deliquoration	Motor Task	Laboratory Instruments	Outcome Measures	Main Findings
Stolze et al., 2000 [28]	10 NPH (75.9 ± 6.3) 20 HS (74.6 ± 5.9)	Tap test (30 mL)	24 h (1 day)	Free-speed locomotion on a 13 m walkway	Imprinted footmarks on a draft paper through punched-out peaks under shoe soles; infra-red movement analysis system (reflective markers on legs)	Stance, swing, and double-limb support phase duration; gait velocity; stride length; cadence; step width; foot angle; step height; stride length CV; step width CV; hip, knee and ankle joint movements	Compared to HS: prolonged stance and double-limb support phases; shorter swing phase; lower gait velocity, stride length, and cadence; higher step width, outward foot rotation, and stride length CV; lower CV of step width, step height, and foot angle; lower maximal knee joint extension. Tap test effects: decreased stance and double-limb support phase duration; increased gait velocity and stride length; decreased stride length CV. No significant clinical–behavioural correlations.
Stolze et al., 2001 [18]	11 NPH (76 ± 6) 12 HS (74.6 ± 5.9) 10 PD (66.4 ± 6.7)	Tap test (30 mL)	24 h (1 day)	Free-speed locomotion on a 13 m walkway, with and without external cues and/or therapy (TAP test for NPH/L-Dopa for PD)	Imprinted footmarks on a draft paper through punched-out peaks under shoe soles; infra-red movement analysis system (reflective markers on legs)	Stance, swing, and double-limb support phase duration; gait velocity; stride length; cadence; step width; foot angle and foot angle CV; step height; stride length CV; hip, knee, and ankle joint movements	Compared to HS: higher stride length CV, longer stance and double-limb support phase duration. Compared to HS and PD: lower gait velocity, stride length, step height, step width variability, foot angle CV, and joint range of motion; higher step width. Tap test effects: decreased stance and double-limb support phase duration; longer swing phase duration; increased gait velocity and stride length; decreased stride length CV. No significant effects of external cues on gait in NPH.
Williams et al., 2008 [12]	15 NPH (73 ± 8) 9 HS (69 ± 7)	CSF drainage (10 mL/h); shunt surgery	72 h of continuous CSF drainage; NA after shunt surgery	Free-speed locomotion on a walkway	Pressure-sensitive carpet system	Double-limb support phase duration; gait velocity; stride and step length; cadence; base width; stride length CV; “functional ambulation profile”	Drainage and shunt surgery effects: increased gait velocity and cadence; decreased double-limb support phase duration. Additional effects of shunt surgery: increased stride length and functional ambulation profile; decreased base width and stride length CV. The degree of response to CSF drainage correlated to the shunt surgery response for double-limb support phase duration, gait velocity, stride length, and cadence.
Allali et al., 2013 [29]	27 NPH (77 ± 10) 22 nNPH (74.5 ± 9)	Tap test (40 mL)	2.10 (1.49) days	Free-speed locomotion on a 10 m walkway during single- and dual-task conditions	In-shoe transducers (footswitches); optoelectronic system (reflective markers on feet)	Stance duration; gait velocity; stride length and time; step width and height	Compared to nNPH: Tap test effects: greater improvement in stance duration, step width, gait velocity, and stride length in NPH than nNPH. Step width as most discriminative parameter. Gait parameters as discriminative measures only during dual-task condition.
Agostini et al., 2015 [14]	60 NPH (73 ± 8) 50 HS (71 ± 12)	Tap test (30–50 mL)	2 to 4 h	Free-speed locomotion on a 9 m pathway	Three footswitches; knee goniometer	Swing, double-limb support, heel contact, flat-foot contact and push-off durations; gait velocity; dynamic knee range of motion; Mahalanobis distance of parameters	Compared to HS: increased double support and flat-foot contact duration; decreased gait velocity, swing phase duration and knee range of motion. Tap test effects: increased gait velocity (also in those who were not candidates for shunt surgery). Mahalanobis distance decreased in tap test responders (the lower the value, the better the performance).
Yang et al., 2016 [30]	50 NPH (78.8 ± 5.5)	CSF drainage (10–15 mL/h)	Daily for 3 days	TUG test	Two inertial sensors placed on the legs	Gait velocity; stride length; cadence	Deliquoration effects: prominent improvement in gait velocity and stride length and, to a lesser extent, cadence and TUG times in one-third of patients. Gait parameters were similar in patients who improved and those who did not after deliquoration.
Schniepp et al., 2017 [31]	24 NPH (76.1 ± 7.8)	Tap test (30–50 mL)	1–8 h; 24 h; 48 h; 72 h	Locomotion on a 6.7 m pathway (preferred, maximal velocity) during single- and dual-task conditions	Pressure-sensitive carpet system	Gait velocity	Tap test effects: increased gait velocity after 24–72 h for all conditions (both single and dual task). Possible false-negative results within the first 24 h. Positive correlations between Evan index and gait velocity improvement, post-drainage gait velocity improvement (48–72 h), and benefit from shunt surgery or repeated CSF subtractions. Dual-task effects: decreased gait velocity compared to single task.
Allali et al., 2017 [32]	68 NPH (75.9 ± 7.4)	Tap test (40 mL)	24 h (1 day)	TUG test with and without dual-task condition	Optoelectronic system	Gait velocity; stride time and width; heel height	Tap test effects: increased gait velocity and heel height, and reduced stride time during both single- and dual-task conditions. Similar walking speed in patients with NPH and NPH mimics.
Kitade et al., 2018 [33]	12 NPH (76.3 ± 4.6)	Shunt surgery	NA	Free-speed locomotion along a 10 m pathway	Optoelectronic system; force plates	Gait velocity; step length; cadence; hip, knee, and ankle joint angles, moment and power (sagittal plane)	Shunt surgery effects: increased gait velocity; step length; hip, knee, and ankle range of motion; and hip peak flexion moment during the stance phase. Positive correlations between the rate of improvement in the Evans index and kinematic changes.
Nikaido et al., 2018 [19]	23 NPH (76.9 ± 4.7) 18 HS (74.3 ± 3.4)	Tap test (NA); shunt surgery	72 h after tap test; 1 week after shunt surgery	Free-speed locomotion along a 10 m pathway	A triaxial accelerometer on the waist (L3)	Gait velocity; number of steps; step time; medio-lateral and vertical center-of-mass movements; CV of step time and movement trajectory amplitudes	Compared to HS: lower gait velocity, higher number of steps, step time, and CV of step time and movement trajectory amplitudes; center of mass movements increased in the medio-lateral axis and decreased in the vertical direction. Tap test and shunt surgery effects: improvement in all considered parameters.
Panciani et al., 2018 [34]	52 NPH (68–84) 300 HS (over 70)	Tap test (40–50 mL)	2 h	Free-speed locomotion along a 10 m pathway	An inertial sensor on the waist	Gait cycle; stance, swing, single, and double support phase durations; gait velocity; cadence; stride length; % stride length/height	Compared to HS: lower swing and single support phase durations, gait velocity, cadence, stride length, % stride length/height; higher gait cycle stance and double support phase durations. Tap test effects: improvement in all parameters. Gait velocity, stride length, and double support duration improved more in responders than non-responders.
Selge et al., 2018 [35]	27 NPH (72 ± 8.1) 38 HS (68.9 ± 7.6) 38 PSP (69 ± 6.3)	NA	NA	6.7 m free-speed locomotion during single task, cognitive dual task, and motor dual task	Pressure-sensitive carpet system	Gait velocity; cadence; step width; stride length; CV of step width, stride time, and length	SINGLE TASK: Compared to HS and PSP: lower gait velocity and stride length; higher step width. Compared to HS: lower cadence and CV of step width; higher CV of stride time and length. DUAL TASK: Compared to HS and PSP: lower gait velocity and stride length; higher step width. Compared to HS: lower cadence and step width; higher CV of stride time and length. Compared to PSP: lower CV of stride time. Lower sensitivity to dual task in NPH than PSP.
Bovonsunthonchai et al., 2018 [36]	27 NPH (77.3 ± 6.92)	Tap test (30–50 mL)	24 h	3 m TUG test	Pressure-sensitive carpet system	Time for sit-to-stand, 3 m walk, 180-degree turn; number of turning steps	Tap test effects: reduced sit-to-stand and walking times; lower number of turning steps.
Song et al., 2019 [37]	28 NPH (75.2 ± 7.3)	Tap test (30–32 mL); shunt surgery	3–4 h after tap test; 6 months after shunt surgery	6 m walking test	Pressure-sensitive carpet system	% Stance; % swing; % single support; cadence; gait velocity; step and stride length; stride width; toe in/out angle degrees; ambulation time	Tap test and shunt surgery effects: higher gait velocity, step, and stride length. Additional effects of shunt surgery: lower % stance and ambulation time; higher % swing, % single support, and gait velocity after shunt surgery than tap test.
Yamada et al., 2019 [38]	28 NPH (77.5 ± 5.9) 87 HS (NA) 29 PWO (NA)	Tap test (30–40 mL); shunt surgery	1 and 4 days	3 m iTUG; 10 m walking test	A smartphone on the abdomen	Acceleration in three axial directions on the 3D scatter plots and their 95% confidence ellipsoid; derived iTUG score	Tap test and shunt surgery effects: shortened iTUG times; increased mean 95% confidence ellipsoid volumes and iTUG scores.
Nikaido et al., 2019 [39]	63 NPH (77.9 ± 5.5)	NA	NA	10 m walking test	An accelerometer on the waist (L3)	CV of step time and movement trajectory amplitude (i.e., center of mass movements) in the ML and VT directions	Positive correlation between the number of falls and the amount of gait variability (i.e., CV of step time and movement trajectory amplitude).
Ishikawa et al., 2019 [40]	32 NPH (77.6 ± 5.5) 87 HS (79.4 ± 7)	Tap test (30 mL); shunt surgery	24 h after tap test; 1 week after shunt surgery	10 m TUG	A smartphone on the abdomen	Times of the TUG components (arising from the chair, straight walking, turning around, walking back, turning back again, sitting down)	Compared to HS: higher times of straight walking, walking back, turning back again, and sitting down components. Shunt surgery effects: improvement in TUG components time.
Giannini et al., 2019 [41]	76 NPH (74.8 ± 4.7)	Tap test (30–50 mL); shunt surgery	24 and 72 h after tap test; 6 months after shunt surgery	TUG test; 18 m walking test	3D instrumented treadmill (force plates)	Number of steps; gait velocity; cadence; stride length	Tap test and shunt surgery effects: decreased number of steps; increased gait velocity, cadence, and stride length. Main effect of the tap test at 72 h.
Lim et al., 2019 [42]	23 NPH (73.0 ± 7.0) 17 HS (69 ± 5.1)	Tap test (30–50 mL)	24 h	5.8 m walking test	Pressure-sensitive carpet system	Stance and swing phase duration; gait velocity; cadence; stride length and time; step width; toe in/out angle; CV of stride time and length	Compared to HS: higher stance phase duration with increased double-limb support; lower gait velocity and stride length; higher step width, toe-out angle, CV of stride time and length. Tap test effects: higher gait velocity and stride length; lower step width and CV of stride time and length. Negative correlation between CV of stride time and length and frontal assessment battery scores in NPH.
Colella et al., 2019 [43]	84 NPH (77.1 ± 6.4)	NA	NA	Free-speed locomotion on a trail	Optoelectronic system	Gait repeatability (for gait deviation index, velocity, cadence, cycle time, stride length, double and single support)	The gait deviation index and stride length show the best repeatability and lowest variability.
Bäcklund et al., 2020 [44]	4 NPH (73 ± 3.2) 87 HS (70)	Shunt surgery	NA	20 m walking test	Two IMUs and an optical infrared distance-triangulating sensor on the legs	Step width; CV of step width	Compared to HS: larger step width. Shunt surgery effects: reduced step width and CV of step width.
Ferrari et al., 2020 [16]	76 NPH (75 ± 4.7)	Tap test (30–40 mL)	24 and 72 h	TUG test; 18 m walking test	Three inertial sensors on the shoes and lower trunk	Single and double support duration; gait velocity; stride length; cadence; total time; number of steps	Tap test effects: reduced double support time and number of turning steps; increased stride length and cadence at 24–72 h, and total time at 72h.
Wolfsegger et al., 2021 [15]	21 NPH (70; 63–80)	Tap test (30–50 mL); shunt surgery	2–4 h after tap test; 3 months after shunt surgery	10 m walking test	A pressure system measuring mobile insoles; markless motion-capture system	Double support phase; gait velocity; step length, height, and variability; hip, knee, and ankle range of motion	Tap test and shunt surgery effects: increased gait velocity, step length, and height, hip, knee, and ankle range of motion.
Morel et al., 2021 [17]	77 NPH (76.1 ± 6.2) with different gait types	Tap test (NA)	24 h	10 m walking test	Optoelectronic system	Gait velocity	Tap test effects: increased gait velocity, especially in patients with frontal gait; patients with parkinsonian gait did not show gait velocity changes.
He et al., 2021 [45]	20 NPH-r (73.5 ± 5.8) 16 NPH-nr (69.6 ± 5.9) 20 HS (NA)	cLD (10 mL/h for 48 h);	4 h	2 min walking test	Six inertial sensors on wrists, feet, sternum, and waist	Double support phase; cadence; gait velocity; stride length; foot strike and toe-out angle; lateral step variability; coronal range of lumbar motion; turning steps; elevation at mid-swing	Compared to HS: reduced cadence, gait velocity, mid-swing elevation, foot strike angle, lateral step variability, stride length, and coronal range of lumbar motion, and increased double support phase, toe-out angle, and turning steps. After CSF drainage (NPH-r vs NPH-nr): greater improvement in cadence, gait speed, double support phase, foot strike angle, stride length, and turning steps in NPH-r than NPH-nr.
Hnin et al., 2021 [46]	27 NPH (76.8 ± 5.5)	NA	NA	TUG test	Video-based motion analysis	Gait velocity; cadence; step length and time; stride length and time; early step length and time; sit-to-stand time, 3 m walking time; turning time; turning step	Patients were evaluated before and after a specific rehabilitation program (action observation). After the intervention, there were significant improvements in step time, early step time, gait speed, sit-to-stand time, and turning time.
Yamada et al., 2021 [47]	97 NPH (76.9 ± 7.3) 68 HS (NA)	Tap test (30–40 mL)	1 and 4 days	10 m walking test	A smartphone on the abdomen	Fluctuations in trunk tri-axial accelerations; derived trunk acceleration index; volume of the 95% confidence ellipsoid for 3D plots of chronological changes of tri-axial accelerations	The outcome measures were correlated to a calculated NPH-specific pathological gait index (based on clinical evaluation). Forward/vertical acceleration fluctuations and trunk acceleration were significantly associated with the probability of an NPH-specific pathological gait. The AUC-ROC curves for detecting an NPH-specific pathological gait were >80% for all investigated parameters
Sun et al 2021 [48]	6 NPH (77.7 ± 5.9) 8 HS (71.5 ± 6.7)	Tap test; shunt surgery	8, 24, and 72 h after tap test; 1 month after surgery	10 m walking test	Wireless force insoles and wearable inertial sensors (only for gait velocity quantification)	Gait velocity; cadence; step time; percentage of double support and stance phase; variability in plantar pressure in gait cycles	Compared to HS: higher plantar pressure variability at baseline and 24/72 h after tap test. Deliquoration effect: only the plantar pressure variability significantly improved 8 h after tap test and 1 month after surgery.
Bäcklund et al., 2021 [49]	4 NPH (73 ± 3.2) 83 HS (70)	Shunt surgery	NA	20 m walking test	Two IMUs and an optical infrared sensor triangulating distance on the legs	Heel height and heel-height variability	Compared to HS: lower heel height and higher heel-height variability before surgery. After surgery, the heel height increased and the variability decreased.
Kuruvithadam et al., 2021 [50]	12 NPH (76 ± 5.4) 20 HS (24.9 ± 2.7) 20 HS (75 ± 8.4)	NA	NA	10 m walking test; 72h home monitoring	Five inertial sensors on both ankles, wrists, and chest	Stance, swing, and double support phase; stance/swing ratio; gait velocity; stride length, time, and time variance; step width; cadence; foot max clearance and outward rotation; number of turning steps; arm swing velocity and amplitude	Compared to HS: increased stance/swing ratio and double support phase (both only in ecological setting); reduced swing and stance phase (only in ecological setting), gait velocity, stride length, and stride time variance (only in experimental setting), foot max clearance, number of turning steps, arm swing velocity, and amplitude; Experimental vs ecological setting: laboratory parameters showed lower stride length, step width, gait velocity, foot max clearance, swing phase, and stride time variance, plus increased foot outward rotation, step width, stance phase, double support phase and stance/swing ratio. The ML algorithms trained on the obtained data were able to classify pathological gait with a high level of accuracy (>90%).
Gago et al., 2022 [51]	8 NPH (77 ± 6.7)	Tap test (40 mL); shunt surgery	2 h after tap test; 6 months after shunt surgery	20 m walking test	Two inertial sensors on both feet	Stride length; gait velocity; liftoff angle; maximum heel height; maximum late toe swing; strike angle; double support phase	Tap test effects: increased stride length, maximum heel height, maximum late toe swing, strike angle, and double support phase. Shunt surgery effects: increased gait velocity, stride length, liftoff angle, maximum heel hight, maximum late toe swing, and strike angle. Gait velocity, stride length, liftoff angle, and maximum heel height negatively correlated with cognitive score (CDR).
Ferrari et al., 2022 [52]	42 NPH (75.2 ± 4.0)	Tap test (30 mL); shunt surgery	72 h after tap test; 6 months after shunt surgery	TUG test; 18 m walking test	Three inertial sensors on the shoes and upper trunk	TUG section times (standing, walking, turning, sitting), number of steps; number of turning steps; cadence; stride length and time; gait velocity; double support phase; phase coordination index; trunk inclination; maximum and minimum foot clearance in different step phases; sagittal foot inclination; frequency of maximum foot motion energy; foot motion energy dispersion	Shunt surgery effects: increased maximum and minimum foot clearance in different step phases, gait velocity, foot motion energy dispersion, stride length, cadence, and frequency of maximum foot motion energy. All TUG section times, all numbers of steps, stride time, double support phase, and phase coordination index were reduced. Clinical scores (Tinetti balance, Tinetti gait, Tinetti total, GSS, and Rankin Scale) showed strong correlation with most of the experimental parameters.
Möhwald et al., 2022 [53]	55 NPH (72.6 ± 4.7) 55 HS (70.5 ± 7.6)	Shunt surgery	NA	Walking test on eight conditions (different speed, during cognitive/motor dual tasks, with eyes closed or head reclination)	Pressure-sensitive carpet system	Stride length and time; stride length and time CV and asymmetry; gait velocity; percentage of swing and double support phases; swing phase CV and asymmetry; stride width; stride width CV	During preferred walking speed, the most significant gait parameter thresholds to identify NPH patients were stride length ≤ 1.02 m (sensitivity 0.93/specificity 0.91/AUC 0.96), gait velocity ≤ 0.83 m/s (0.80/0.91/0.93), double support phase ≥ 27.0% (0.96/0.76/0.91), and stride length coefficient of variation ≥ 3.4% (0.93/0.72/0.90).
Lotan et al., 2022 [54]	82 NPH 36 NPH-r (79.3 ± 6.3) 46 NPH-nr (77.2 ± 6.1)	HVLP or cLD	Immediately after, 24 h after, and 72 h after CSF removal	5.8 m walking test	Pressure-sensitive carpet system	Functional ambulation performance score (FAP)	NPH-r vs NPH-nr: increased percentage change in FAP at all post-CSF drainage evaluations. Correlation between temporal horns volume and gait velocity improvement in NPH-r.
Nikaido et al., 2022 [55]	20 NPH-hfr (79.2 ± 5.7) 20 NPH-lfr (77.7 ± 4.7) 23 HS (75.7 ± 4.4)	NA	NA	5 m walking test	Optoelectronic system	Gait velocity; cadence; step length and width; percentages of the gait cycle time (stance, swing, single support, and total double support)	Compared to HS: slower gait velocity, shorter step length, and wider step width. NPH-hfr vs NPH-lfr: NPH-HFR group had significantly slower gait velocity, shorter step length, and wider step width.
Iseki et al., 2023 [56]	23 NPH (77.0 ± 6.4) 23 PD (70.1 ± 6.0) 92 HS (72.3 ± 6.3)	NA	NA	Walking in a 1 m circle for 1–3 laps, clockwise (cw) and counter-cw	Motion capture with a smartphone application (TDPT-GT)	Fluctuation index of body segments	Compared to HS: significant fluctuations were found in all body segments in NPH and PD patients during walking. Compared to PD: fluctuations during walking were prominent in the NPH group.
Cakmak et al., 2023 [57]	13 NPH (71.9 ± 4.1) 20 PD (69.1 ± 6.9)13 HS (69.2 ± 9.0)	NA	NA	TUG test; 10 m walking test; 2 min walking test	Three inertial sensors on the shoes and lower trunk	Stance, swing, and double support phase duration; gait velocity; cadence; stride length and time; foot strike angle; toe in/out angle; lateral step variability; midswing elevation; circumduction; TUG section durations	Compared to HS and PD: reduced gait velocity, stride length, swing phase, toe off angle. Increased double support and stance phase. Compared only to HS: decreased foot strike ankle. Compared only to PD: increased TUG total duration and turn duration, and reduced TUG turn velocity.
Dias et al., 2023 [58]	11 NPH (77 ± 6.7) 20 HS (74 ± 8.6)	Shunt surgery	3 to 6 months after shunt surgery	10 m walking test; 72 h home monitoring	Five inertial sensors on both ankles, wrists, and chest	Stance, swing, and double support phase; stance/swing ratio; gait velocity; stride length, time, and time CV; step width; cadence; foot max clearance and outward rotation; number of turning steps; arm swing velocity and amplitude	Compared to HS: increased step width, number of turning steps, and stride time CV. Reduced stride length, gait velocity, max foot clearance, cadence (only with pre-shunt patients), arm swing velocity, and arm swing amplitude (only with pre-shunt patients). Shunt surgery effects: increased swing phase. ROC analysis revealed the cutoff stride length of ≥0.44 m and gait velocity of ≥0.39 m/s as predictors for good VPS responsiveness.
Mori et al., 2023 [59]	70 NPH (75.5 ± 5.8) 20 HS (75.1 ± 5.1)	NA	NA	1 min walking test under three conditions: normal, fast, and during cognitive dual task	Pressure-sensitive carpet system	Stride length, width, and time; step length and time; gait velocity; cadence; percentages of gait cycle time (stance, swing, single support, and total double support)	Normal and fast walking: increased double support phase, stride width, step time, and stance phase; reduced cadence (only during normal walking), gait velocity, stride length, step length, swing, and single-support phase. Dual tasking: increased double-support phase, stride width, and stance phase; reduced gait velocity, stride length, swing, and single-support phase. All experimental parameters correlated with the BBS and SPPB.
Bovonsunthonchai et al., 2024 [60]	51 NPH (78.3 ± 6.3) 23 NPH-r (77.4 ± 7.1) 28 NPH-nr (79.1 ± 5.6)	Tap test (30–50 mL)	24 h after tap test	5 m walking test (3 m on platform)	Pressure-sensitive carpet system synchronized with a camera	Foot rotation angle; step length, time, and width; stride length; cadence; gait velocity, and percentage of gait phases: stance, loading response, single-limb support, pre-swing, swing, double-limb support	Tap test effects: improvement in step length, stride length, step time, stride time, cadence, and gait velocity. NPH-r vs NPH-nr: Tap test responders showed significant improvements in right step length and time, stride length and time, cadence, and gait velocity.
Na et al., 2024 [61]	9 NPH (76 ± 5.3) 14 HS (34 ± 11.8)	NA	NA	15 m walking test under three conditions: normal, fast, and during cognitive dual task	Sixteen inertial sensors on head, upper and lower trunk, pelvis, upper arms, forearms, hands, thighs, shanks, and feet	ROM of the shoulder (sagittal), hip (sagittal and transversal), knee (sagittal), and ankle (transversal); ankle maximum plantarflexion; stride time	Reduction in all ROMs compared to HS.

AUC-ROC: areas under the receiver-operating characteristic curves; BBS: Berg Balance Scale; CDR: Clinical Dementia Rating scale; CM: cervical myelopathy; CSF: cerebrospinal fluid; CV: coefficient of variation; cLD: continuous lumbar drain; GSS: Gait Status Scale; HS: healthy subjects; HVLP: high-volume lumbar puncture; iTUG: instrumented timed up-and-go test; LST: lumbar stenosis; ML: machine learning; NA: not available; NoS: number of subjects; nNPH: patients with normal pressure hydrocephalus-like conditions; NPH: patients with normal pressure hydrocephalus; NPH-r and -nr: patients with normal pressure hydrocephalus responsive and unresponsive to deliquoration; NPH-hfr and -lfr: patients with normal pressure hydrocephalus with high and low fall risk; PD: patients with Parkinson’s disease; PSP: patients with progressive supranuclear paralysis; PWO: patients with diseases other than normal pressure hydrocephalus; ROM: range of motion; SPPB: Short Physical Performance Battery; TUG: timed up-and-go test; VPS: ventriculoperitoneal shunt.

**Table 2:** Kinematic measures of gait in normal pressure hydrocephalus.

Kinematic Measure		VS Age-Matched HS	Deliquoration Effects
Gait cycle	Swing phase	↓=	↑
	Stance phase	↑	↓
	Single-limb support	↓=	↑
	Double-limb support phase	↑	↓
Cadence		↓=	↑
Velocity		↓	↑
Stride	Length	↓	↑
	Time	=	↓
	Width	=↑	↓
	CV time	↑	↓
	CV length	↑	↓
Step	Length	↓	↑
	Time	↑=	↑
	Width	↑	↓
	Height	↓	↑
	CV time	↓	↑
	CV width	↓	↓
Foot angle (toe in-out angle)		↑	=
Shoulder, hip, knee, and ankle ROM		↓	↑
TUG duration		↑	↓

CV: coefficient of variation, HS: healthy subjects, NPH: patients affected by normal pressure hydrocephalus, ROM: range of motion, TUG: timed up-and-go test, ↑: increased, ↓: reduced, =: unchanged.

**Table 3:** Instrumental evaluation of balance in normal pressure hydrocephalus.

Study	NoS	CSF Drainage	Assessment Time After CSF Subtraction	Motor Task	Instruments	Outcome Measures	Main Findings
Blomsterwall et al., 2000 [20]	17 NPH (66 ± 14) 10 SAE (73 ± 3) 23 HS (67 ± 13)	Shunt surgery	3 months after shunt surgery	Upright stance (feet together, heels together, tandem, on one leg, EO/EC)	Force platform	AP/ML COP displacement; sway area; AP/ML displacement ratio; forward/backward sway velocities; COP inclination in the sagittal plane	Compared to HS: higher ML and AP displacements, higher sway area and backward velocity with eyes open; lower AP/ML ratio; higher eyes open/closed ratio. Compared to SAE: higher AP displacement; lower AP/ML ratio and backward velocity when standing with feet together and eyes closed. Shunt surgery effects: decreased sway area and AP displacement when standing with feet together and eyes open; improved inclination.
Czerwosz et al., 2009 [62]	9 NPH (50–84) 47 HS (50–69)	Shunt surgery	NA	Upright stance (EO/EC)	Force platform	Sway area; sway radius	Compared to HS: increased sway radius and area in both conditions; EC did not worsen the performance. Shunt surgery effects: reduced sway radius and area in both conditions; EC worsened the performance.
Blomsterwall et al., 2011 [63]	20 NPH (65 ± 10) 11 HS (51 ± 15)	NA	NA	Upright stance (EO/EC)	Force platform	AP/ML COP displacements; AP/ML displacement ratio; AP/ML sway velocities	Compared to HS: increased ML displacement with EC and AP displacement with EO and EC. Higher AP/ML sway velocities.
Szczepek et al., 2012 [64]	57 patients (64 ± 13): 18 NPH + 36 BA 47 HS (59.9 ± 7)	NA	NA	Upright stance (EO/EC)	Force platform	Sway radius; sway area	Compared to HS: higher sway radius and sway area with EO and EC. Compared BA: higher sway radius and sway area only with EO. Conversely to HS and BAs, among NPH the postural performance did not change between EC/EO tasks.
Czerwosz et al., 2013 [65]	18 NPH (64 ± 13) 36 BA (64 ± 13) 47 HS (60 ± 7)	Shunt surgery	1 week after shunt surgery	Upright stance (EO/EC)	Force platform	Sway area; sway radius; length of COP displacement	Compared to HS and BA: higher sway radius, sway area, and COP displacement with EC and EO. EC did not worsen the postural performance. Shunt effects: improvement in all parameters. EC worsened the postural performance. Statistical classification accurately distinguished between NPH before and after surgery, and between NPH and BAs.
Lundin et al., 2013 [66]	35 NPH 73 (49–81) 16 HS 73 (62–89)	Shunt surgery	3 months after shunt surgery	Upright stance	Computerized dynamic posturography	AP sway in different conditions, quantified as SOT scores	Compared to HS: lower SOT scores. Shunt surgery effects: higher composite SOT score.
Abram et al., 2016 [67]	17 NPH (75.7 ± 7.04)	Tap test (30–40 mL)	NA	Upright stance	Computerized dynamic posturography	AP sway in different conditions, quantified as SOT scores	Tap test effects: higher composite SOT, SOT 2, SOT 4, and SOT 5 scores; significant improvement in somatosensory and visual performance.
Bäcklund et al., 2017 [68]	31 NPH (78 ± 8) 22 VM (69 ± 10) 58 HS (71 ± 4)	Tap test (NA); cLD; shunt surgery	4 h after tap test; 3 months after shunt surgery	Upright stance (normal, tandem, semi-tandem, feet together, EO/EC, on foam support); walking under different conditions (10 m, 10 m over barriers, 6 m on foam)	An inertial sensor (i.e., gyroscopes) on the waist (L3-L4)	AP/ML sway angles; AP/ML sway velocities	Compared to HS: higher sway angle and sway velocity in all directions during upright stance; lower sway angles and velocities with EC; lower sway velocity during gait. Compared to VM: similar measures except for lower sway velocity during gait. Tap test and shunt surgery effects: reduced AP sway angle during upright stance; increased sway angles with EC; reduced sway velocity during gait.
Nikaido et al., 2018 [69]	23 NPH (76.9 ± 4.7) 18 HS (75.6± 4.1)	Tap test (NA); shunt surgery	72 h after tap test; 1 week after shunt surgery	Upright stance; voluntary multi-directional leaning	Force platform	Locus length; sway area; AP/ML COP displacement during maximal voluntary leaning; stability area	Compared to HS: increased locus lengths and sway areas during quiescent standing. During multidirectional leaning, increased locus lengths and sway area; reduced AP and ML maximal displacements. Reduced stability area. Shunt surgery effects: Increased AP and ML maximal displacements during multidirectional leaning; increased stability area.
Nikaido et al., 2018 b [19]	27 NPH (76.9 ± 4.5) 20 PD (72.3 ± 5.6) 20 HS (75.6 ± 4.1)	NA	NA	Upright stance; voluntary multi-directional leaning	Force platform	Locus length; sway area; locus length per unit area; AP/ML COP displacement during maximal voluntary leaning; stability area	Compared to HS: increased locus length and sway areas during quiescent standing. During multidirectional leaning, increased locus lengths and sway areas in each direction; reduced AP and ML maximal displacement and stability area. Compared to PD: increased sway area and locus length per unit area during ML leaning; reduced stability area.
Heß et al., 2021 [21]	12 NPH (74.6 ± 4.1) 18 NPH after shunt (72.0 ± 7.0) 20 HS (71.5 ± 3.6)	NA	NA	Upright stance	Force platform	95% COP confidence area; AP/ML COP displacement; AP/ML sway velocity	Compared to HS: higher values for all investigated COP parameters and broader stability area. Shunt surgery effects: no significant changes.
Wolfsegger et al., 2021 [15]	21 NPH 70 (63–80)	Tap test (30–50 mL); shunt surgery	2–4 h after tap test; 3 months after shunt surgery	Upright stance	An inertial sensor on the waist	Sway area	Tap test and shunt surgery effects: no significant changes in trunk sway area.
Nikaido et al., 2022 [55]	20 NPH-hfr (79.2 ± 5.7) 20 NPH-lfr (77.7 ± 4.7) 23 HS (75.7 ± 4.4)	NA	NA	5 m walking test	Optoelectronic system	AP/ML COM-BOS distance; AP/ML sway velocities; AP/ML margin of stability	Compared to HS: reduced AP COM-BOS distance and AP COM velocity. Increased ML COM-BOS distance and ML COM velocity, larger AP and ML MOS. NPH-hfr vs NPH-lfr: NPH-HFR group exhibited significantly shorter AP and longer ML COM-BOS distance, slower AP COM velocity, and larger ML MOS.
Daly et al., 2022 [70]	21 NPH (72.6 ± 7.6), stratified on UPDRS pull test response 20 HS (70 ± 4.0)	cLD (3 days)	After cLD removal (4th day)	Pull test	Fifteen inertial sensors on head, upper trunk, pelvis, upper arms, forearms, hands, thighs, shanks, feet	COM velocity; COM acceleration; reaction time; step length	COM velocity profile differed between patient groups. Patients with worse clinical response showed reduced COM peak velocity and later peak velocity onset. NPH patient groups differed in both the ability to scale step length to increasing pull intensity and the overall step length for a given intensity. Reaction time and step length scaling distinguishes NPH patients from HS.
Cakmak et al., 2023 [57]	13 NPH (71.9 ± 4.1), 20 PD (69.1 ± 6.9), 13 HS (69.2 ± 9.0)	NA	NA	Upright stance	Three inertial sensors on the shoes and lower trunk	Sway area; AP/ML COP displacement; AP/ML sway velocities; root mean square (RMS) sway; path length; jerk	Compared to HS: increased RMS sway, AP/ML velocities, and AP/ML displacements. Compared to PD: increased sway area, RMS sway, and ML displacement.
Park et al., 2024 [71]	56 NPH (75.5 ± 5.5)	Tap test (30–50 mL)	24–48 h after tap test	Upright stance	Force platform	AP/ML sway velocities; root mean square COP (rmsCOP); turns index; torque; BOS area; spectral density of COP oscillation in AP/ML directions (both peak and average)	Tap test effects: reduced sway velocities, rmsCOP, turn index, torque, BOS area, AP/ML average, and peak spectral density at 0–0.5.
Na et al., 2024 [61]	9 NPH (76 ± 5.3) 14 HS (34 ± 11.8)	NA	NA	Upright stance (EO/EC)	Force platform	AP force distribution (%); AP/ML sway; overall COP sway (or passed COP distance)	Compared to HS: increased AP sway, all parameters worsened with EC.

AP: antero-posterior; BAs: patients with brain atrophy; BOS: base of support; cLD: continuous lumbar drain; COM: center of mass; COP: center of pressure; EC: eyes closed; EO: eyes open; NA: not available; NoS: number of subjects; NPH: patients with normal pressure hydrocephalus; NPH-hfr and -lfr: patients with normal pressure hydrocephalus with high and low fall risk; ML: medio-lateral; PD: patients with Parkinson’s disease; SAE: subcortical arteriosclerotic encephalopathy; SOT: sensory organization test; UPDRS: Unified Parkinson’s Disease Rating Scale; VMs: patients with ventriculomegaly.

**Table 4:** Kinematic measures of balance in normal pressure hydrocephalus.

Kinematic Measure	VS Age-Matched HS	Deliquoration Effects
COP AP displacement	↑	↓
COP ML displacement	↑	↓
Mean COP displacement	↑	↓
AP COM sway velocity	↑	↓
ML COM sway velocity	↑	↓
Mean COM velocity	↑	NA
Sway area	↑	↓
Stability area	↑	↓

AP: antero-posterior, COP: center of pressure, HS, healthy subjects, ML: medio-lateral, NA: not available, ↑: increased, ↓: reduced, =: unchanged.

## Data Availability

Not applicable.

## Supplementary Materials

The following supporting information can be downloaded at: https://www.mdpi.com/article/10.3390/bioengineering12020135/s1, Table S1: PRISMA checklist; Table S2: Quality assessment.
